# The accuracy of the Stroke Risk Analysis (SRA) system for predicting atrial fibrillation in patients in the postoperative period of myocardial revascularization

**DOI:** 10.1371/journal.pone.0282565

**Published:** 2023-03-15

**Authors:** Kleber Rogério Serafim, Dalmo Antônio Ribeiro Moreira, Paulo Alexandre da Costa, Ricardo Garbe Habib, Carlos A. Sierra Reyes, Cláudia da Silva Fragata

**Affiliations:** Instituto Dante Pazzanese de Cardiologia, São Paulo, Brasil; Policlinico Casilino, ITALY

## Abstract

**Introduction:**

Postoperative myocardial revascularization atrial fibrillation (POAF) is a clinical complication that affects about 30% of patients and its mechanisms of origin are still poorly understood. This fact makes it difficult to identify the patient at greatest risk for this arrhythmia. This mission seems evident due to the complications it entails, including longer hospital stays, risk of stroke, heart failure, and death. There are reports of preoperative clinical aspects inherent to the patient’s condition, such as gender and age, and discontinuation of beta-blockers as risk factors. In addition, additional information obtained by electrocardiogram, echocardiogram, and blood count data, for example, present only modest predictive results. The analysis of heart rate and heart rate variability obtained by the Stroke Risk Analysis System (SRA) is a technique used to predict ambulatory atrial fibrillation (AF), using recordings of only one hour showing good accuracy. This system, however, has not yet been used to predict the emergence of POAF. The rationale for its use is based on the suspicion that the emergence of POAF is strongly related to sympatho-vagal imbalance and the increase in atrial ectopia, that is, changes in heart rhythm, the main variables analyzed by the SRA algorithm.

**Objective:**

To assess the accuracy of the SRA to identify patients at risk of having POAF after coronary artery bypass graft surgery (CABG).

**Method:**

114 consecutive patients with coronary artery disease underwent coronary artery bypass grafting between the years 2015 and 2018. Between the first and fifth postoperative days, they underwent continuous electrocardiographic monitoring using the Holter system for cardiac rhythm analysis. Patients were divided into two groups: Group I was formed of those with POAF and Group II included patients without POAF. The tracings obtained by Holter were reanalyzed using the CardioManager®/Cardios program, converted and divided into one-hour sections using the SRA®/Cardios and Geratherm Converter program and submitted to the SRA-Apoplex medical/Geratherm® analysis algorithm. The SRA identifies three possibilities for classifying patient risk: a) Risk 0: patient in sinus rhythm; b) Risk 1: patient at increased risk for paroxysmal AF; c) Risk 2: patient with AF already present. For Group I, SRA were considered positive when Risks 1 and 2 were identified. For Group II, those identified as Risk 0 were considered negative SRA.

**Results:**

POAF occurred in 33/114 patients (28%). The sensitivity, specificity, positive predictive value, and negative predictive value of the SRA to identify patients with POAF were 69%, 84%, 69%, and 82%, respectively; the positive and negative likelihood ratios, in addition to the accuracy of the SRA were, respectively, 4.3%, 0.36%, and 79%. A subanalysis of the results of the day on which AF occurred was performed on the records obtained in the first three hours of recording and up to three hours before the appearance of POAF. In the first period, the SRA was able to predict POAF in 57% of cases, while in the second period, the system identified the arrhythmia in 83% of cases.

**Conclusions:**

a) The SRA presents good accuracy to predict POAF; b) its accuracy is moderate in the first three hours of recording; c) the accuracy increases significantly near the beginning of POAF; d) these findings indicate that electrophysiological changes that precede POAF are acute, occurring a few hours before the event and are identified by the SRA algorithm.

## Introduction

Postoperative atrial fibrillation (POAF) after cardiac surgery is a complication that occurs in 25 to 40% of patients after coronary artery bypass graft surgery (CABG) [1] and in more than 60% of patients undergoing combined procedures [2]. It usually manifests within the first five days and has the highest incidence between the second and third days. It can occur both in the paroxysmal form and with longer episodes, without spontaneous reversion. POAF usually does not last very long, as sinus rhythm is spontaneously restored in 90% of cases within 8 weeks [1,2].

In general, the main risk factors related to its appearance are history of atrial fibrillation (AF), patient age, mitral stenosis, heart failure, anemia, metabolic changes, and tissue inflammation. Regarding the surgical procedure, the type of cardioplegia, cardiopulmonary bypass time, hypoxia, postoperative pericarditis, and sympathetic hyperactivity complete the list [3–5].

POAF is associated with worsening hemodynamic status and increased perioperative and intraoperative morbidity and mortality, such as thromboembolic events (stroke) and congestive heart failure [4]. Stroke is more likely in patients with higher CHA_2_DS_2_VASc scores. In addition to the aforementioned risks, there is also an increase in the length of hospital stay and, consequently, an increase in hospital costs [6,7]. In the late postoperative period, patients with POAF have a higher risk of death from all causes and, in this condition, this may be a marker of cardiovascular risk for future events [6]. For these reasons, efforts should be made regarding the early identification of patients at higher risk.

Information related to patients such as gender and age, discontinuation of beta-blockers, in addition to electrocardiogram, echocardiogram, and even blood count data (neutrophil/lymphocyte ratio, for example) have been used to identify patients in the preoperative period. risk for POAF, but with only modest results.

Stroke Risk Analysis (SRA) is an electrocardiographic monitoring system that allows the identification of risk of AF in outpatients. The system is equipped with specific software that analyzes different components of the heart rate, its regularity, as well as the presence of atrial ectopia. Clinical studies have demonstrated the high accuracy of this system for identifying patients at risk of outpatient AF [8–10]. The role of the SRA in the identification of patients at risk for POAF has not yet been evaluated in our country.

The aim of this study was to determine the accuracy of the SRA for identifying the risk of POAF in patients undergoing coronary artery bypass graft (CABG) surgery.

## Methodology

The research project was approved by the Research Ethics Committee of Instituto Dante Pazzanese de Cardiologia (IDPC), in session of May 21, 2018, under opinion 2,664,947. The consent term adopted from the participants was written and the patients who agreed to participate in the research had to sign it.This was a prospective longitudinal observational analytical study, in which the outcome was the occurrence of POAF up to the fifth postoperative day after CABG.

The medical records of patients over 18 years of age, with coronary artery disease undergoing isolated CABG at Instituto Dante Pazzanese de Cardiologia (IDPC), between 2015 and 2018 were included. Patients undergoing combined surgeries were excluded, as were patients with the presence of atrial flutter or AF in the immediate postoperative period, those with a previous history of AF or paroxysmal atrial flutter, and patients with cardiac pacemakers.

### Study protocol

In the preoperative period, clinical data, in addition to electrocardiogram (ECG) and echocardiogram data, were evaluated and analyzed. Postoperatively, the patients underwent continuous electrocardiographic monitoring from the first to the fifth postoperative day using Holter monitoring (CardioSeven®/Cardios). The recordings were analyzed using the CardioManager®/Cardios program, converted and divided into one-hour segments using the SRA®/Cardios and Geratherm Converter program, and submitted to the SRA-Apoplex medical/Geratherm® analysis algorithm. Holter recording was not started in the immediate postoperative period due to greater clinical instability at this stage and, consequently, greater risk of removing the device for procedures and surgical re-approach, among others.

### Stroke Risk Analysis (SRA) system

**Fig 1:**
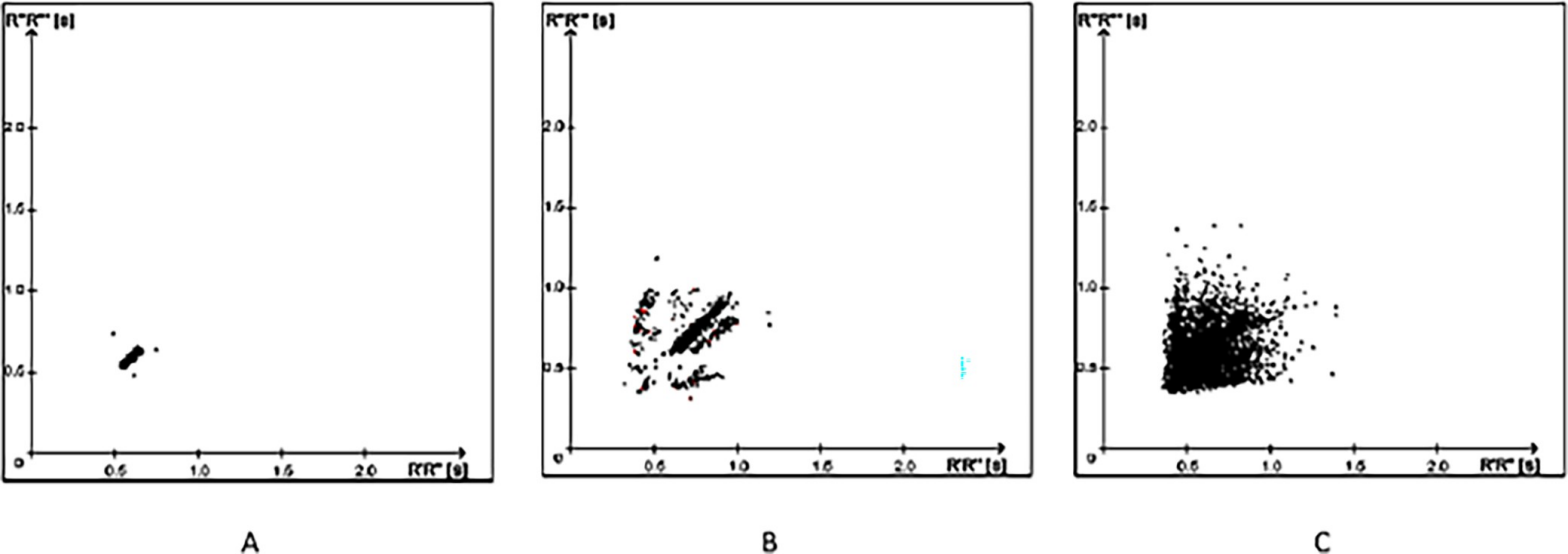
Poincaré plots based on RR intervals on Holter tracings. On the abscissa are the values of the preceding RR intervals and on the ordinate are the current RR intervals. The shape of the distribution of points on the graph visually defines the three conditions in which the patient may be. In A, clustered points or on a stick or golf club indicate that the patient is in sinus rhythm; in B, scattered points along the rod indicate a patient at risk of atrial fibrillation; in C, the full dispersion of points indicated that the patient is in atrial fibrillation (see discussion in text).

The SRA has an algorithm that analyzes the influences of the autonomic nervous system (ANS) on the heart, using linear and non-linear parameters of heart rate variability (HRV) in the time domain to differentiate between sinus rhythm, paroxysmal AF, and persistent AF. The main component of this system is the Poincaré plot, for which analysis can be done qualitatively (visually) and quantitatively. The qualitative form described by Tulppo et al. [11], as illustrated in **Fig 1**, is performed by analyzing the plot classified according to the image formed by the dispersion of points [12].

In addition to visual analysis, the SRA uses an electrocardiographic analysis algorithm to assess the risk of developing AF. To obtain the Poincaré plot, the fluctuations and dynamics of the RR intervals are evaluated. Instead of plotting an RR interval against its previous one, the differences between two consecutive RR intervals are plotted and normalized by the formula: [8]

**{R_I_−(R_I_ + 1)/R_I_ + (R_I_ + 1)}**

**Fig 2:**
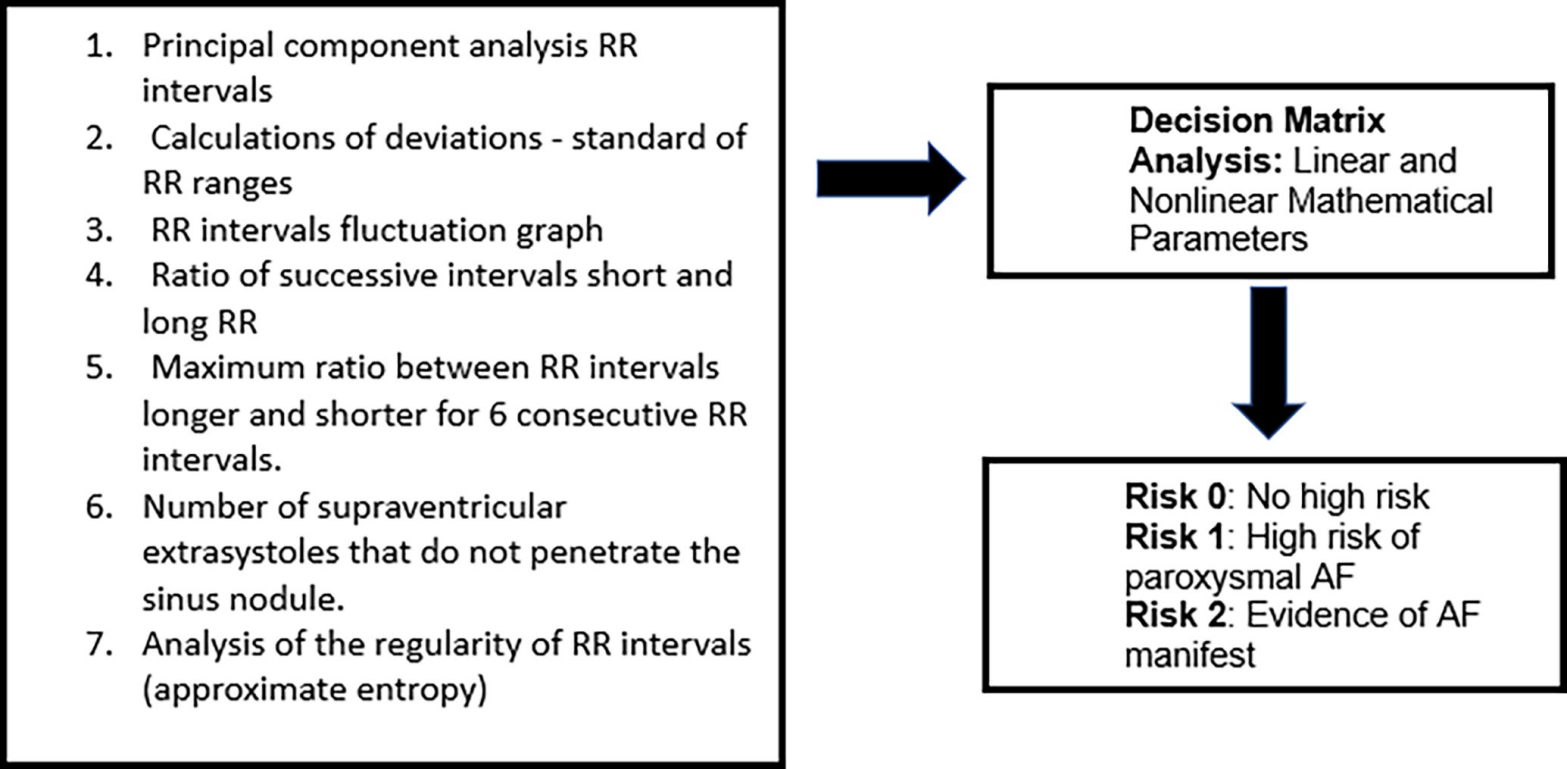
Components of the algorithm for electrocardiographic analysis used by the SRA.

The other component is part of the SRA decision matrix, still based on the HRV. In this case, statistical methods (mean and standard deviation) are used for temporal variations in milliseconds or by percentage of variations of consecutive cycles. Therefore, through mathematical parameters, it is possible to establish the risk of AF and maximize the discriminatory accuracy between three groups, as shown in **Fig 2**(adapted from Duning et al., 2011) [8].

The linear and non-linear parameters evaluated are based on the variability of heart rate in the time domain.

Parameters 2,4,5 and 6 are considered linear parameters. Parameters 1,3 and 7 are nonlinear parameters.

### SRA assessment in the detection of POAF

Patients were divided into two groups: Group I was formed of those with POAF with a minimum duration of 30 seconds and Group II included patients without POAF.

In patients who had POAF, recordings of up to 24 hours prior to the onset of POAF were selected. These recordings were divided into one-hour segments and submitted to the SRA-Apoplex medical/Geratherm® analysis algorithm. For patients in Group II, that is, those without AF documented by 24-hour Holter, tracings from the second postoperative day were selected, a criterion chosen because it was the day with the highest incidence of POAF in Group I. If positive, the SRA result that presented Risk 1 or 2, in each period of one hour, is shown in Fig 1. When Risk 0 was identified, the patient had no documented AF. In patients in Group II, a Risk 1 or 2 record was considered a false positive result.

In Group I patients, the first three hours of recording and the three hours before POAF were evaluated. In turn, in the control group, the first three and the last three times of recording obtained in the tracings of the second postoperative day were evaluated.

**Fig 3:**
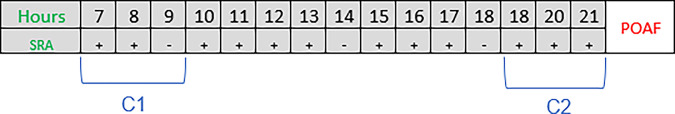
Example of a patient with POAF at 9 pm on the first postoperative day. Again, the predominance of positive results that characterize the patient at risk for POAF.

**Fig 4:**
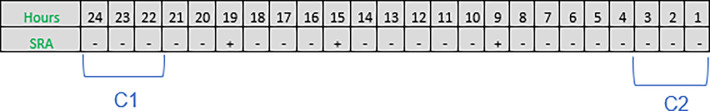
Example of a patient in the group II whose tracings were obtained on the second postoperative day. This patient has a greater number of negative results and only three positive results (false positives).

**Fig 5:**
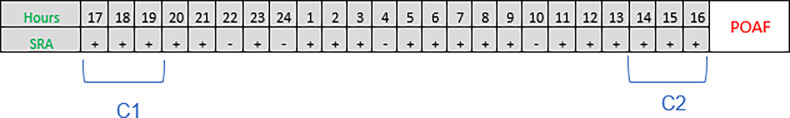
Example of a patient with POAF at 16:00 on the second postoperative day. Note that in this patient there is a predominance of positive results, characterizing a patient at risk for POAF.

Figs 3 and 4 illustrate, in the first line, the hours before the event, identified as POAF (in red at the end of the line), while the second line indicates the positive result of SRA [+] indicating the highest probability of risk of POAF; SRA [–] indicates that the system did not detect, at that time, the risk criteria for POAF. C1 corresponds to the evaluation of the first three hours of recording and C2 the three hours that preceded POAF. Fig 5 depicts what happened to a patient in the control group (distribution similar to the previous figures). Note that, at most time points, the SRA did not identify criteria to classify the presence of POAF ([–]), while at some time points, the classification indicated Risk 1 for POAF (false positive result).

## Statistical analysis

To calculate the sample size, a statistical power of 90% and a significance of 5% was considered. Based on previous studies, whose accuracy of the SRA was to identify paroxysmal AF in outpatients or in stroke units, a sensitivity and specificity of 80% was considered. The prevalence of FAPO was considered to be 30%, the same found in the analysis of the database that gave rise to the present study.

Taking into account the data described, the initial sample size was 94 individuals. However, a loss of approximately 10% was estimated and, for this reason, it was decided to increase the minimum sample size to 104 individuals.

Quantitative variables are presented as mean and standard deviation and qualitative variables as absolute frequency and percentage. To analyze whether there were differences between groups in quantitative variables, the Student t-test or the Mann-Whitney test was used, depending on the assumption of normality, when the intention was to measure the discriminative power of the quantitative variables. For qualitative variables, Fisher’s exact or the chi-squared test was used. Values of P<0.05 were considered significant.

The accuracy of SRA was calculated based on 2x2 contingency tables. The following variables were determined: sensitivity, specificity, positive predictive value, and negative predictive value, as well as positive and negative likelihood ratios.

## Results

### Characteristic of the study population

A total of 123 patients undergoing CABG between 2015 and 2018 were included. Of these, 9 patients were excluded after reviewing the medical records due to combined surgery, evidence of paroxysmal AF and acute AF in the intraoperative or immediate postoperative period or in the first 6 hours after the beginning of Holter monitoring, thus totaling 114 patients who were part of this sample. In Group I, 21 patients received intravenous amiodarone for reversal of POAF and 11 patients had spontaneous reversal. No patient required electrical cardioversion Table 1 lists the characteristics of the population studied in Groups I and II. There was a predominance of FAPO in males (74.3%) with a mean age of 61+/-8 years (ranging from 43 to 84 years). The population’s mean CHA_2_DS_2_VASc score was 4.2, with 4.31 for Group I and 4.16 for Group II (P = NS). Age was higher in Group I (65.1 years) versus Group II (59.7 years) (p<0.001). Beta-blocker withdrawal was more frequent in patients in Group I when compared to patients in Group II (p = 0.02). No significant differences were found between Group I and Group II with regard to cardiovascular risk factors, echocardiographic variables, and variables related to the surgical procedure.

**Table 1:** Characteristics of the studied population.

	Group I (n = 32)	Group II (n = 82)	*p*-value
Age (years, sd)	65.1(6.2)	59.7(8)	**<0.001**
Male (n, %)	28(87.5)	78(95.1)	0.05
Arterial hypertension (n, %)	30(93.7)	71(86.5)	0.18
Diabetes (n, %)	16(50)	38(46.3)	0.83
Smoking (n, %)	6(18.7)	27(32.9)	0.28
Previous stroke (n, %)	3(50)	3(50)	0.35
Beta blocker use (n, %)	30(93.7)	60(73)	**0.02**
CHA_2_DS_2_VASC (n, sd))	4.31(0.78)	4.16(0.89)	0.37
BMI kg/m^2^ (n, sd)	27.3(4)	28(4.6)	0.3
Ejection fraction (%, sd)	51.7(10.5)	54.4(12.2)	0.06
Left atrium diameter (mm, sd)	41.1(5.3)	39.9(4.7)	0.22
Left ventricle diameter (mm, sd)	54.09(5.7)	53.1(7.1)	0.17
Aortic root diameter (mm, sd)	35.6(3.6)	34.1(4.7)	0.06
Perfusion time (min, dp)	80.72(25.3)	74.3(23.1)	0.51
Anoxia time (min, dp)	51.7(13.2)	48.7(15.9)	0.58

SD: Standard deviation, n: Number, BMI: Body mass index, mm: Millimeter, min: Minutes.

### Occurrence of FAPO

POFA occurred in 32 patients (28%) with the highest incidence documented on the second postoperative day. There was no predominance in the time for POAF’s manifestation.

### Relationship between the autonomic nervous system POAF

When analyzing the HRV components of the first hour of holter recording and the last hour immediately before POAF, a significant increase in the high frequency (HF) component was noted, a fact not observed in the low frequency (LF) component. Consequently, there was a drop in the LF/HF ratio.

### Assessment of the SRA in the detection of FAPO

Holter tracings selected from the 32 patients in Group I totaled 660 one-hour stretches, while in Group II, they totaled 1,288 stretches. The SRA identified 458 stretches as a risk of AF in Group I (positive result) and in Group II it ruled out this possibility in 1,082 stretches (negative result). Sensitivity (Sens), specificity (Esp), positive predictive value (VP+), negative predictive value (VP-), accuracy (Ac), positive likelihood ratio (RV+), and negative likelihood ratio (RV-), were respectively 69%, 84%, 69%, 82%, 79%, 4, 3, and 0.36, as shown in Table 2.

**Table 2:** Detection of FAPO risk in Groups I and II by the SRA. The numbers correspond to the total of positive and negative stretches in each group.

Exam		Group I			Group II			Total	
**SRA (+)**		**458**			**206**			**664**	
**SRA (-)**		**202**			**1082**			**1284**	
**Total**		**660**			**1288**			**1948**	
**Sens**	**Esp**		**VP+**	**VP-**		**Accuracy**	**RV+**		**RV-**
**69.3%**	**84%**		**68.9%**	**84.2%**		**79%**	**4,3**		**0,36**

### SRA assessment in FAPO prediction

When the first three hours of recording for all patients were analyzed, the number of positive results was 57% in Group I patients versus 22% in Group II (*P* < 0.001). When analyzing the three hours that preceded POAF in Group I and compared with the last three hours of patients in Group II, the number of positive results was 83% and 11%, respectively (P = 0.000) (Table 3).

**Table 3:** SRA (+) analysis of the first three hours of recording and the three hours before POAF in all patients.

	Group I	Group II	*p*
**First three hours of recording**	**57%**	**22%**	**<0,001**
**Three hours before POAF**	**83%**	**11%**	**,000**
**Difference**	**26%**	**11%**	
***p***	**0,003**	**0,13**	

There was a 26% increase in positive SRA results in Group I when the first three hours of recording were compared with the three hours before POAF (*P* = 0.003). Group II, on the other hand, showed no significant change in the results of positive SRA when comparing the results in the first three hours of recording with the last three on the second postoperative day.

When analyzing the SRA results in the first three hours of recording, FAPO occurred in 56% of patients who had two or more positive results. Of those in whom the first three hours had one or no positive RAS result, only 15.4% had POAF. The results in this scenario show a sensitivity of 61.3% and specificity of 81.4%, PV+ 56%, and PV- of 84% of the RAS to predict POAF (**Table 4**).

**Table 4:** Total number patients with two or more positive SRA results (Risk 1) in Groups I and II in the initial three hours of recording.

Exam		Group I		Group II		Total	
**SRA (≥ 2+)**		**19**		**15**		**34**	
**SRA (< 2+)**		**12**		**66**		**78**	
**Total**		**31**		**81**		**112**	
**Sens**	**Esp**		**VP+**		**VP-**		**Accuracy**
**61.3%**	**81.4%**		**55.8%**		**84.6%**		**75.9%**

When analyzing the SRA results of patients who had two or more positive results in the three hours before POAF, AF occurred in 65% of patients in group I. Those who had one or no positive SRA results in this period, only 7% had POAF. The results in this scenario show an SRA sensitivity of 84%, specificity of 83%, PV+ of 65%, and PV- of 93% (Table 5).

**Table 5:** Total number of patients with two or more positive SRA results (Risk 1) in Groups I and II in the three hours preceding POAF (Group I) or three hours of recording on the second postoperative day (Group II).

Exam		Group I		Group II		Total	
**SRA (≥ 2+)**		**26**		**14**		**40**	
**SRA (< 2+)**		**5**		**67**		**72**	
**Total**		**31**		**81**		**112**	
**Sens**	**Esp**		**VP+**		**VP-**		**Accuracy**
**83.8%**	**82.7%**		**65%**		**93%**		**83%**

There was loss of electrocardiographic signals in two patients, which explains the smaller number of patients compared to the initial sample. In neither Group I or Group II was Risk 2 documented, i.e. AF present.

## Discussion

This study demonstrates that the SRA accurately identifies POAF, as demonstrated for the identification of AF in outpatients. The sensitivity, specificity, positive predictive value, and negative predictive value were, respectively, 69%, 84%, 69%, and 82%. Furthermore, it was demonstrated that the degree of accuracy was higher (89%) within the three-hour period preceding the onset of POAF, thus indicating that this arrhythmia has its own peculiarities, different from AF in outpatients, particularly due to the greater intensification of autonomic activity near the onset of the arrhythmia.

Several studies have evaluated the role of the SRA in the identification of patients with paroxysmal AF in outpatient settings or in ischemic stroke units [13–15]. However, this is the first study that has evaluated the SRA as a predictor of POAF after CABG. AF is the most common arrhythmia after cardiac surgery and its prevention is a major challenge today, aiming to reduce the risk of complications and the length of hospital stay.

### Role of the SRA in the identification and prediction of FAPO

The use of the recording system for only one hour showed good accuracy for the prediction of AF in outpatients. It is not certain whether the same would apply to the assessment of POAF due to the different characteristics of the clinical behavior of this arrhythmia and the risk factors for it being triggered. In outpatients, the factors that generate and maintain AF are present for a longer time, in already remodeled atria, with electrical circuits already established, depending only on factors that destabilize the arrhythmogenic substrate for its emergence. In this study, however, in addition to the patients not having documented AF before surgery, left atrial remodeling was not present since its diameter was normal in both groups. This is an important difference from ambulatory AF.

### Influences of the autonomic nervous system and the prediction of POAF by the SRA

The SRA assesses the characteristics of sinus rhythm in terms of regularity, linear variability of heart rate in the time domain through mean and standard deviation indices, and the analysis of the qualitative and quantitative form of Poincaré graphs, in addition to the presence of atrial ectopia. Therefore, it is able to predict the possibility of AF depending on the characteristics of these risk variables. The influence of the sympathetic and parasympathetic systems as well as intrinsic atrial neural activity is determined by this analysis technique.

Recent studies have evaluated interference of the autonomic nervous system as a predictor of POFA. Park et al. [16] evaluated a possible autonomic imbalance as a predictor of POAF through heart rate turbulence. This technique evaluates short-term fluctuations in the sinus rate after a ventricular extrasystole, using two indices: onset of turbulence (TO or turbulence onset) and slope of turbulence (TS or turbulence slope). Changes in these indices are independent predictors for POAF, in addition to being associated with worse long-term outcomes, such as the recurrence of AF and stroke. This technique, however, implies that the patient has ventricular ectopia with sufficient density to apply its algorithm.

Despite the controversial autonomic influence determined by the analysis of classical heart rate variability, in the time and frequency domains at the origin of FAPO, in contrast to what happens with the analysis of heart rate turbulence, some authors have demonstrated that the competitive coactivation of the sympathetic and parasympathetic systems could be responsible for the emergence of this arrhythmia, rendering it impossible to determine the predominant role of one or the other in the origin of the arrhythmia [17].

Xiong et al. [18] demonstrated, when following up patients undergoing CABG, that the electrophysiological changes that preceded AF occurred approximately one hour before the event. Such alterations were characterized by an increase in the density of atrial ectopia, facilitated by an increase in sympathetic tone and, consequently, in heart rate. These variables are captured by the SRA. These findings were demonstrated in this study, when it was observed that the accuracy of the system was higher a few hours before the appearance of POAF. This indicates that the destabilizing factors of atrial electrical activity become more intense close to the onset of AF, supporting the theory that the emergence of POAF occurs due to an increase in the punctual instability of atrial electrical activity at some point in the day, unlike AF of other etiologies in which alterations would be present for a longer time. These findings are also evident when it was demonstrated that the positive results of the SRA in Group I were much lower in the first three hours of recording (far from the documented POAF) in relation to the last three hours before the arrhythmia (increase of 26%; p< 0.003). When the first three hours of recording were evaluated, considering two positive results as a predictor of POAF, the following changes were observed in comparison with the three hours that preceded the arrhythmia: sensitivity increased from 61.3% to 83.8%; specificity from 81% to 83%; positive predictive value from 56% to 65%; negative predictive value from 85% to 93%.

The results of this study corroborate the observations of autonomic imbalance that precedes POAF, and support the information that beta-blockers are useful drugs for its prevention [19,20].

## Study limitations

These results and the contribution of this study to the production of knowledge must be considered within some context limitations. The first limitation is that these results apply only to patients undergoing CABG. Second, the SRA was not used in the immediate postoperative period and this may have caused a loss of FAPO records. The non-use on that day was due to possible intercurrences during this period that could compromise the recordings, forcing the removal of the Holter recorder, making analysis by the SRA unfeasible. The other limitation is due to the interference in the baseline in the Holter tracings which, although not always preventing their analysis, can alter the SRA results, invalidating their analysis and interpretation. However, this did not happen in this study.

## Conclusions

The SRA has modest accuracy for predicting POAF when analyzed in a period that precedes its appearance by more than 3 hours; however, its accuracy is high within the three hours that precede the arrhythmic event, suggesting that the instability of atrial electrical activity is an acute event mediated by the autonomic nervous system.

## References

[1] Crystal E, Connolly SJ, Sleik K, Ginger TJ, Yusuf S. Interventions on Prevention of Postoperative Atrial Fibrillation in Patients Undergoing Heart Surgery: A Meta-Analysis. Circulation. 2002 Jul 2;106(1):75–80. doi: 10.1161/01.cir.0000021113.44111.3e 12093773

[2] Villareal RP, Hariharan R, Liu BC, Kar B, Lee VV, Elayda M, et al. Postoperative atrial fibrillation and mortality after coronary artery bypass surgery. J Am Coll Cardiol. 2004 Mar;43(5):742–8. doi: 10.1016/j.jacc.2003.11.023 14998610

[3] Feldman A, Moreira DAR, Gun C, Wang HTL, Hirata MH, de Freitas Germano J, et al. Analysis of Circulating miR-1, miR-23a, and miR-26a in Atrial Fibrillation Patients Undergoing Coronary Bypass Artery Grafting Surgery: Atrial Fibrillation Patients Undergoing Coronary Bypass Artery Grafting Surgery. Ann Hum Genet. 2017 May;81(3):99–105. 28422282 10.1111/ahg.12188

[4] Mathew JP, Fontes ML, Tudor IC, Ramsay J, Duke P, Mazer CD, et al; Investigators of the Ischemia Research and Education Foundation; Multicenter Study of Perioperative Ischemia Research Group. A multicenter risk index for atrial fibrillation after cardiac surgery. JAMA. 2004 Apr 14;291(14):1720–9. doi: 10.1001/jama.291.14.1720 . 15082699

[5] Dobrev D, Aguilar M, Heijman J, Guichard JB, Nattel S. Postoperative atrial fibrillation: mechanisms, manifestations and management. Nat Rev Cardiol. 2019 Jul;16(7):417–436. doi: 10.1038/s41569-019-0166-5 30792496

[6] Schwann TA, Al-Shaar L, Engoren MC, Bonnell MR, Goodwin M, Schwann AN, et al. Effect of new-onset atrial fibrillation on cause-specific late mortality after coronary artery bypass grafting surgery†. Eur J Cardiothorac Surg. 2018 Aug 1;54(2):294–301. 29481591 10.1093/ejcts/ezy028

[7] Aranki SF, Shaw DP, Adams DH, Rizzo RJ, Couper GS, VanderVliet M, et al. Predictors of Atrial Fibrillation After Coronary Artery Surgery: Current Trends and Impact on Hospital Resources. Circulation. 1996 Aug;94(3):390–7. 8759081 10.1161/01.cir.94.3.390

[8] Duning T, Kirchhof P, Wersching H, Hepp T, Reinhardt R. Extended Electrocardiographic Poincare Analysis (EPA) for Better Identification of Patients with Paroxysmal Atrial Fibrillation. J Clin Exp Cardiol [Internet]. 2011 [cited 2022 Jun 29];02(02).

[9] Rizos T, Güntner J, Jenetzky E, Marquardt L, Reichardt C, Becker R, et al. Continuous Stroke Unit Electrocardiographic Monitoring Versus 24-Hour Holter Electrocardiography for Detection of Paroxysmal Atrial Fibrillation After Stroke. Stroke. 2012 Oct;43(10):2689–94. doi: 10.1161/STROKEAHA.112.654954 22871678

[10] Soliman EZ, Cammarata M, Li Y. Explaining the inconsistent associations of PR interval with mortality: The role of P-duration contribution to the length of PR interval. Heart Rhythm. 2014 Jan;11(1):93–8. doi: 10.1016/j.hrthm.2013.10.003 24096163

[11] Tulppo MP, Mäkikallio TH, Seppänen T, Laukkanen RT, Huikuri HV. Vagal modulation of heart rate during exercise: effects of age and physical fitness. Am J Physiol-Heart Circ Physiol. 1998 Feb 1;274(2):H424–9. doi: 10.1152/ajpheart.1998.274.2.H424 9486244

[12] Brennan M, Palaniswami M, Kamen P. Poincaré plot interpretation using a physiological model of HRV based on a network of oscillators. Am J Physiol-Heart Circ Physiol. 2002 Nov 1;283(5):H1873–86. 12384465 10.1152/ajpheart.00405.2000

[13] Soliman EZ, Cammarata M, Li Y. Explaining the inconsistent associations of PR interval with mortality: The role of P-duration contribution to the length of PR interval. Heart Rhythm. 2014; 11, 93–98. doi: 10.1016/j.hrthm.2013.10.003 24096163

[14] Rizos T, et al. Continuous stroke unit electrocardiographic monitoring versus 24-hour Holter electrocardiography for detection of paroxysmal atrial fibrillation after Stroke. 2012 Oct;43(10):2689–94. doi: 10.1161/STROKEAHA.112.654954 22871678

[15] Duning T, Kirchhof P, Wersching H, Hepp T, Reinhardt R. Extended Electrocardiographic Poincare Analysis (EPA) for Better Identification of Patients with Paroxysmal Atrial Fibrillation. J. Clin. Exp. Cardiol. 2011; 02(02):02–07.

[16] Park SJ, On YK, Kim JS, Jeong DS, Kim WS, Lee YT. Heart rate turbulence for predicting new-onset atrial fibrillation in patients undergoing coronary artery bypass grafting. Int J Cardiol. 2014 Jul;174(3):579–85. doi: 10.1016/j.ijcard.2014.04.130 24798780

[17] Amar D, Zhang H, Miodownik S, Kadish AH. Competing autonomic mechanisms precedethe onset of postoperative atrial fibrillation. J Am Coll Cardiol. 2003 Oct;42(7):1262–8. doi: 10.1016/s0735-1097(03)00955-0 14522493

[18] Xiong F, Yin Y, Dubé B, Pagé P, Vinet A. Electrophysiological Changes Preceding the Onset of Atrial Fibrillation after Coronary Bypass Grafting Surgery. Chen X, editor. PLoS ONE. 2014 Sep 23;9(9):e107919. doi: 10.1371/journal.pone.0107919 25247814 PMC4172567

[19] Kim SH, Jang M jin, Hwang HY. Perioperative Beta-Blocker for Atrial Fibrillation after Cardiac Surgery: A Meta-Analysis. Thorac Cardiovasc Surg. 2021 Mar;69(02):133–40. 10.1055/s-0040-1708472 32252112

[20] Norhayati MN, Shaiful Bahari I, Zaharah S, Nik Hazlina NH, Mohammad Aimanazrul Z, Irfan M. Metoprolol for prophylaxis of postoperative atrial fibrillation in cardiac surgery patients: systematic review and meta-analysis. BMJ Open. 2020 Oct;10(10):e038364. doi: 10.1136/bmjopen-2020-038364 33130564 PMC7670955

